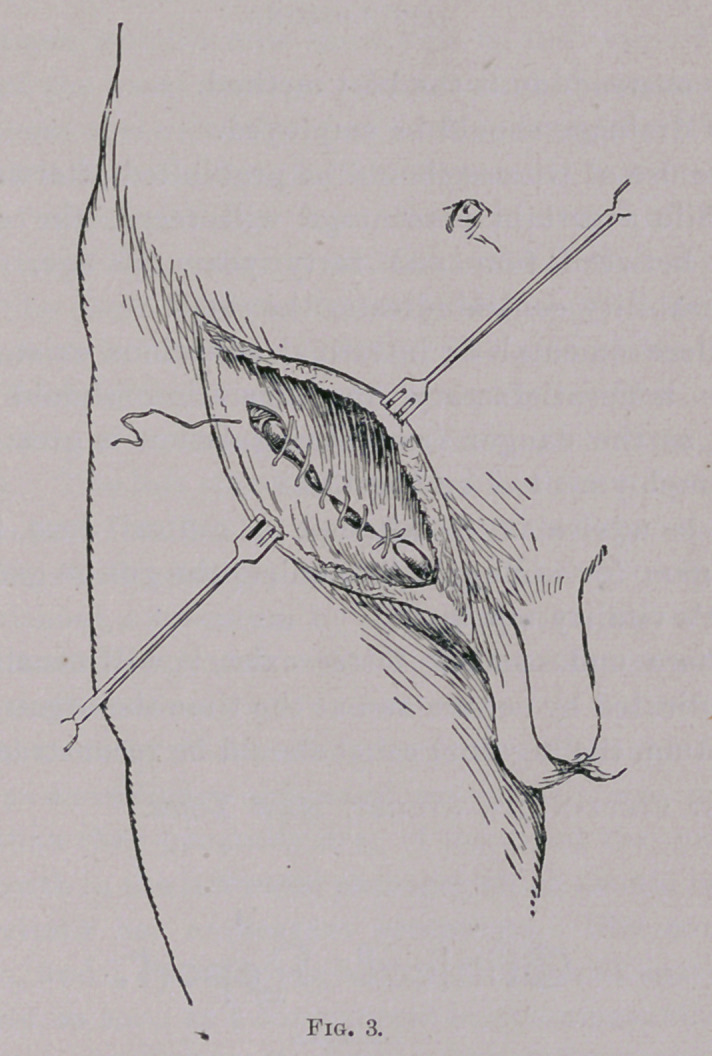# A Review of the Operative Treatment for the Radical Cure of Inguinal Hernia1Read before the Pan-American Medical Congress, in Washington, D. C., September 6, 1893.

**Published:** 1893-10

**Authors:** Samuel E. Milliken

**Affiliations:** Lecturer on Surgery at the New York Polyclinic; 36 West Fifty-Ninth Street, New York


### A REVIEW OF THE OPERATIVE TREATMENT FOR THE RADICAL CURE OF INGUINAL HERNIA.[1]

By SAMUEL E. MILLIKEN, M. D.

Lecturer on Surgery at the New York Polyclinic.

Having but recently given my views on the subject of the Radical Cure for Inguinal Hernia, I wish to discuss only briefly the comparative merits of three methods, viz., that of Bassini, of Kocher, and of Halsted.

After having looked up more than one hundred cases of recurrent hernia after various methods, which came under my observation at the Hospital for Ruptured and Crippled, I am convinced that the percentage is far greater than the operators are led to suppose by the immediate apparent good results. Hospital cases are very difficult to follow, particularly in the large cities, because the poorer classes change their addresses so often, without leaving behind them any tidings as to their whereabouts.

It is my opinion that we expect too much for the radical operation anyway, owing to the fact that, with all of Nature's devices, she has failed to let the structures of the spermatic cord

---

1. Read before the Pan-American Medical Congress, in Washington, D. C., September 6, 1893.

make their exit from the abdominal wall, without leaving each subject liable to hernia—no age being exempt. Our laparotomists will, I think, agree that any abdominal section is likely to be followed by hernia, whatever precautions are taken in closing the wound; and from our herniotomies, when the testicle is not sacrificed, I don't see how we can but expect a larger percentage of recurrences than they get of the ventral variety.

So long as it is not deemed advisable to sacrifice the testicle, the best that we can hope for is to imitate Nature and reëstablish the obliquity of the canal. This is best done, in my opinion, by the method of Bassini; and I will endeavor to show its advantages over the other two methods above mentioned.

FIG. 1.

First, the aponeurosis of the external oblique muscle is divided over the cord structures, until the internal ring is well exposed; the flaps are separated from their underlying structures until the conjoined tendon on the upper, and the shelving process of Poupart's ligament on the lower, are brought into view. The cord structures and the hernial sac are next lifted out of their bed

*en masse*, after which the sac is isolated, opened, and tied off at the highest point.

Secondly, with the cord held out of the operation field by a blunt hook, or the finger of an assistant, the conjoined tendon on the upper is sutured to the shelving process of Poupart's on the lower by means of the kangaroo tendon, or of chromatized catgut,

FIG. 2.

but I prefer the former, as the time for absorption is longer, and the sutures are stronger in proportion to their size. From four to six interrupted sutures will be sufficient to make a firm posterior wall for the cord structures, care being taken not to constrict them at the internal ring.

Thirdly, the obliquity is reëstablished by bringing together the flaps of the external oblique, by a continuous suture of the same material. The skin wound is closed with interrupted catgut without drainage.

Kocher's method simply deals with the sac, which is dissected out as thoroughly as possible, without slitting up the canal, and pulled through a small opening, which he makes in the aponeurosis of the external oblique, opposite the internal. He afterwards sutures it to the aponeurosis, over the canal, but in so doing only strengthens the outer wall.

FIG. 3.

Halsted's method is simply the transplantation of the cord without any particular endeavor to rebuild the canal. After dividing the external oblique, he lifts out the cord structures, and ties off the sac; then sutures the respective tendons together, posterior to them, allowing them to come out directly instead of obliquely. Although I have never met with any recurrences from this operation, possibly owing to its being rarely employed in New York, I am confident that, sooner or later, a hernia will develop at the point of exit of the cord structures. Another decided objection to this procedure is the liability to adhesions with the overlying fascia, which will not occur when they pass between the serous covered tendons.

Of something over thirty cases, operated upon by me after the method of Bassini, I have had three recurrences. In each, suppuration occurred, and the hernia made its appearance during the first six months.

CONCLUSIONS.

1. Reconstruction is the best method.
2. No drainage should be employed.
3. The use of trusses should be prohibited afterwards.
4. While a certain percentage will recur, the operation is justifiable between four and forty years of age, unless some physical disability contraindicates the same.
5. Where omental or intestinal adhesions exist, and the use of a truss is unsatisfactory, the operation should always be attempted, as the danger from strangulation is greater than that from the employment of an anesthetic.
6. It is advisable to attempt a radical cure in all cases operated upon for strangulation, unless the gut be gangrenous, or the patient's vitality too low.
7. Where undescended testes exist, it will usually be found to be complicated by hernia, and at the time the organ is anchored to the scrotum, the inguinal canal should be reconstructed.

36 West Fifty-Ninth Street, New York.

## Figures and Tables

**Fig. 1. f1:**
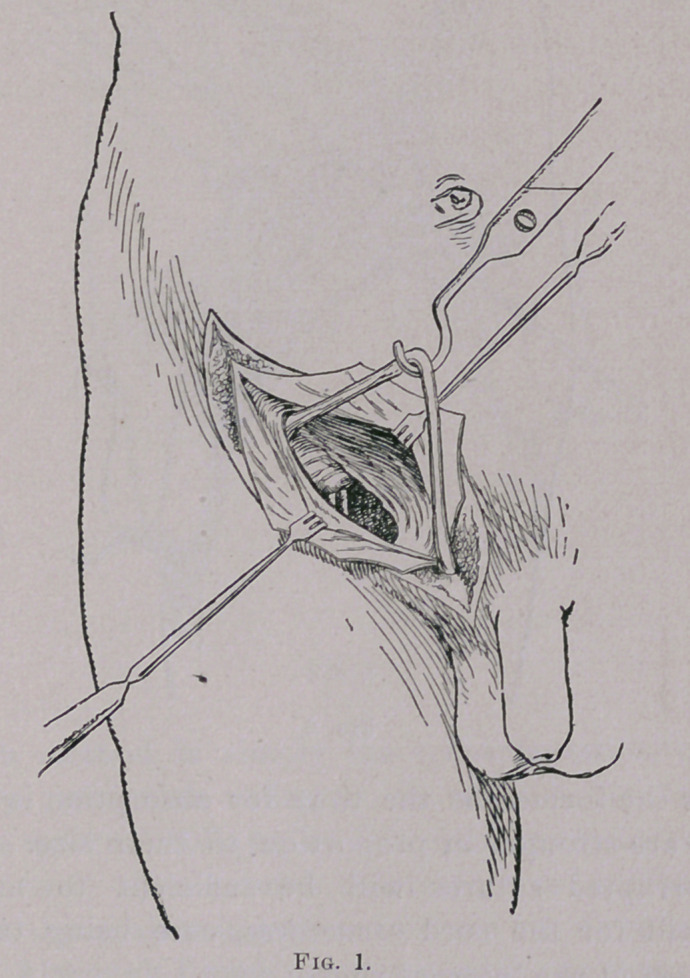


**Fig. 2. f2:**
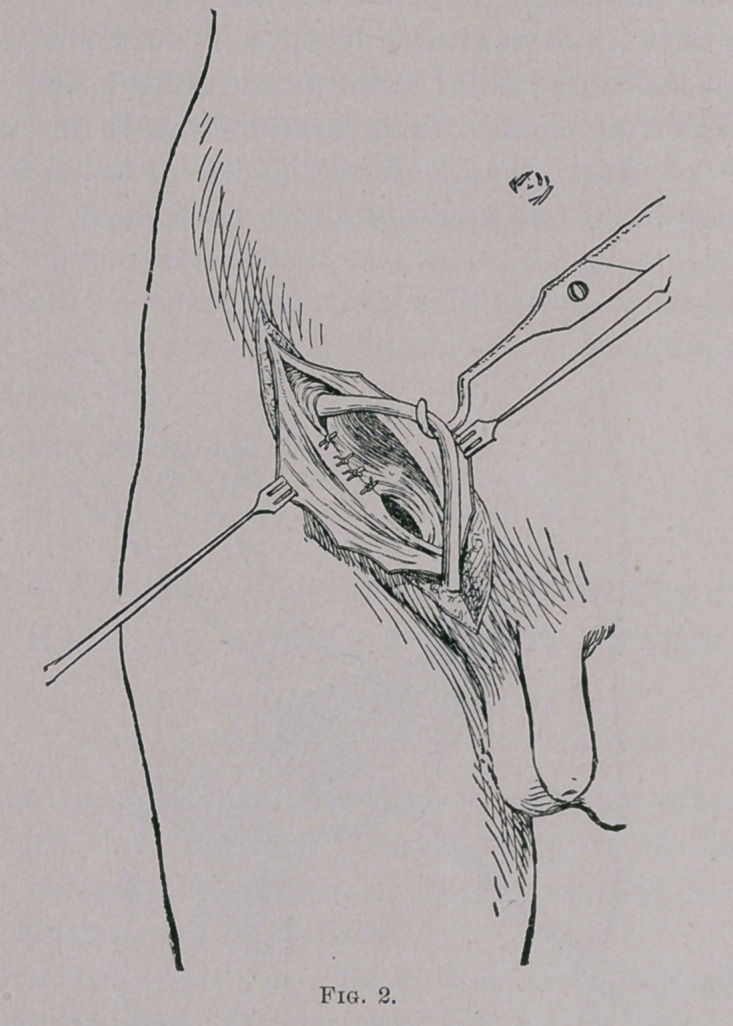


**Fig. 3. f3:**